# Targeting tubulointerstitial remodeling in proteinuric nephropathy in rats

**DOI:** 10.1242/dmm.018580

**Published:** 2015-08-01

**Authors:** Saleh Yazdani, Ryanne S. Hijmans, Fariba Poosti, Wendy Dam, Gerjan Navis, Harry van Goor, Jacob van den Born

**Affiliations:** 1 Department of Medicine, Division of Nephrology, University of Groningen and University Medical Center Groningen, Groningen, The Netherlands; 2 Department of Pathology and Medical Biology, Division of Pathology, University of Groningen and University Medical Center Groningen, Groningen, The Netherlands

**Keywords:** Proteinuria, Renal lymphatic, Lymphangiogenesis, Renal fibrosis, Renal inflammation

## Abstract

Proteinuria is an important cause of tubulointerstitial damage. Anti-proteinuric interventions are not always successful, and residual proteinuria often leads to renal failure. This indicates the need for additional treatment modalities by targeting the harmful downstream consequences of proteinuria. We previously showed that proteinuria triggers renal lymphangiogenesis before the onset of interstitial inflammation and fibrosis. However, the interrelationship of these interstitial events in proteinuria is not yet clear. To this end, we specifically blocked lymphangiogenesis (anti-VEGFR3 antibody), monocyte/macrophage influx (clodronate liposomes) or lymphocyte and myofibroblast influx (S1P agonist FTY720) separately in a rat model to investigate the role and the possible interaction of each of these phenomena in tubulointerstitial remodeling in proteinuric nephropathy. Proteinuria was induced in 3-month old male Wistar rats by adriamycin injection. After 6 weeks, when proteinuria has developed, rats were treated for another 6 weeks by anti-VEGFR3 antibody, clodronate liposomes or FTY720 up to week 12. In proteinuric rats, lymphangiogenesis, influx of macrophages, T cells and myofibroblasts, and collagen III deposition and interstitial fibrosis significantly increased at week 12 vs week 6. Anti-VEGFR3 antibody prevented lymphangiogenesis in proteinuric rats, however, without significant effects on inflammatory and fibrotic markers or proteinuria. Clodronate liposomes inhibited macrophage influx and partly reduced myofibroblast expression; however, neither significantly prevented the development of lymphangiogenesis, nor fibrotic markers and proteinuria. FTY720 prevented myofibroblast accumulation, T-cell influx and interstitial fibrosis, and partially reduced macrophage number and proteinuria; however, it did not significantly influence lymphangiogenesis and collagen III deposition. This study showed that proteinuria-induced interstitial fibrosis cannot be halted by blocking lymphangiogenesis or the influx of macrophages. On the other hand, FTY720 treatment did prevent T-cell influx, myofibroblast accumulation and interstitial fibrosis, but not renal lymphangiogenesis and proteinuria. We conclude that tubulointerstitial fibrosis and inflammation are separate from lymphangiogenesis, at least under proteinuric conditions.

## Abstract (precis)

**Summary:** Targeting lymphangiogenesis, inflammation or fibrosis separately in a rat model of proteinuric nephropathy showed that treating any of these changes alone is not effective in treating the disease.

## INTRODUCTION

Proteinuria is a major challenge in clinical nephrology because sustained proteinuria can lead to a progressive decline in kidney function, worsening to chronic kidney disease (CKD) and end-stage renal disease (ESRD), and eventually the need for dialysis or renal transplantation (Cravedi and Remuzzi, 2013; Ruggenenti et al., 2012; Lea et al., 2005; Gorriz and Martinez-Castelao, 2012). Many renal diseases are accompanied by proteinuria. Because proteinuria is independently associated with a decline in renal function, anti-proteinuric treatment [mainly renin-angiotensin-aldosterone system (RAAS) intervention, eventually in combination with reduced salt intake] makes up a major cornerstone in renal medicine. Nevertheless, complete annihilation of proteinuria is practically not possible, and most patients slowly progress towards renal failure. Forced titration of proteinuria by dual RAAS intervention (ONTARGET trial) or angiotensin-converting enzyme (ACE) inhibition under very-low-salt conditions worsened renal outcomes (Mann et al., 2008) or interstitial fibrosis (Hamming et al., 2006). Even under rather low proteinuria values kidneys deteriorate over time. This indicates the need for additional treatment modalities, not only trying to reduce proteinuria even further, but also reduce the harmful effects downstream of proteinuria (de Zeeuw, 2008). It is well recognized that proteinuria (ultrafiltrated plasma proteins) activates tubular cells to secrete many chemokines and mediators that can elicit proinflammatory and profibrotic cascades (Eddy, 2004; Zoja et al., 2004; Bakris, 2008; Moreno et al., 2014), and leads to renal inflammation and fibrosis (Abbate et al., 2006). An additional treatment option is thus to reduce tubulointerstitial changes secondary to proteinuria.

We previously showed that proteinuria can promote renal lymphangiogenesis that concomitantly occurs with an increase of the profibrotic response and tubular activation (Yazdani et al., 2012). Several studies have proposed both a direct and an indirect link between lymphangiogenesis, inflammation and fibrotic reactions. Nevertheless, their causal interplay in tubulointerstitial remodeling in kidney diseases has not been investigated yet (Yazdani et al., 2014). A wealth of evidence has shown a reciprocal interaction between inflammation and lymphangiogenesis (Johnson and Jackson, 2010; Dieterich et al., 2014). On one hand, leukocytes are able to produce mediators and growth factors that can promote lymphangiogenesis, and, on the other hand, activated lymph endothelial cells (LECs) can secrete several mediators that recruit inflammatory cells and can further exacerbate this inflammatory microenvironment (Loffredo et al., 2014). Among inflammatory cells, macrophages have been shown to play a prominent role in inducing lymphangiogenesis, at least in two distinct ways: by producing lymphangiogenic growth factors and stimuli, and/or by directly trans-differentiating into LECs (Ran and Montgomery, 2012).

**TRANSLATIONAL IMPACT**

**Clinical issue**

Proteinuria (high levels of proteins in the urine) is a major challenge in clinical nephrology. Sustained proteinuria might lead to progressive loss of renal function, which will eventually require dialysis or renal transplantation. Routine anti-proteinuric treatment regimens are not always completely successful, and residual proteinuria might also cause irreversible tissue damage and, ultimately, loss of kidney function. This underlines the urgent need for other therapeutic strategies, not aimed at eliminating residual proteinuria but at preventing proteinuria-induced renal injury, instead. Hence, strategies targeting the downstream effects of proteinuria to preserve kidney function are highly desirable.

****Results****

In this study, the authors used adriamycin-injected rats, a well-established model in which the animals develop proteinuria 6 weeks post-injection. At this stage, lasting for other 6 consecutive weeks, anti-proteinuric treatments started, which targeted specifically three major proteinuria-associated tubulointerstitial changes: lymphangiogenesis (via anti-VEGFR3 antibody), monocyte/macrophage influx (via clodronate liposomes) and interstitial fibrosis (via the S1P agonist FTY720). At week 12, proteinuric rats showed a significant increase of renal lymphangiogenesis, inflammatory cell influx (as evidenced by increased macrophage and T-cell numbers) and fibrosis markers [as evidenced by myofibroblasts accumulation, collagen III deposition and periodic acid-Schiff (PAS) scoring, a measure of interstitial fibrosis]. Anti-VEGFR3 antibody completely inhibited lymphangiogenesis in proteinuric rats; however, it had no significant effect on inflammatory cells, fibrotic markers or proteinuria. Clodronate liposomes reduced macrophage influx and partly prevented myofibroblast increase but showed no effects on renal lymphangiogenesis, fibrotic markers or proteinuria. FTY720 markedly reduced myofibroblast accumulation, T-cell infiltration and interstitial fibrosis (not collagen III deposition), and partially reduced macrophage number and proteinuria, but had no influence on lymphangiogenesis.

****Implications and future directions****

These results suggest that targeting any of the downstream tubulointerstitial changes of protenuria individually is not an effective strategy, at least under proteinuric conditions. Future treatments should aim for combination therapies. Alternatively, because activated tubular epithelial cells are known to mediate tubulointerstitial-tissue remodeling, future strategies could be developed to test whether preventing the activation of tubular epithelial cells in proteinuric conditions or blocking and/or inactivating the chemokines and mediators that these cells secrete can reduce proteinuria-induced renal injury.

The direct link between inflammation and fibrosis has been well established as well (Wick et al., 2013). Despite many clinical and experimental investigations, effective treatment for fibrosis is still lacking in the clinic (Friedman et al., 2013). FTY720, an S1P analog, effectively inhibits the egress of T and B cells from lymph nodes (Kabashima et al., 2006; Matloubian et al., 2004), thereby reducing the number of antigen-primed/restimulated cells that recirculate to peripheral inflammatory tissues (Brinkmann and Lynch, 2002), and consequently halts inflammation. FTY720 can also directly block lymphangiogenesis (Yoon et al., 2008), and has been reported to prohibit renal fibrogenesis (Shiohira et al., 2013; Ni et al., 2013a,b). Taking these all into account, FTY720 seems to be a promising agent in targeting tubulointerstitial remolding. However, the exact interaction among these interstitial phenomena (inflammation, fibrosis and lymphatic remodeling) in proteinuric nephropathy has not been clearly explored. Understanding of the detailed mechanisms of the complex interaction between these proteinuria-induced downstream tubulointerstitial events might reveal their significance to the progression towards ESRD, and hence might have potential therapeutic values in affected individuals.

To mimic a clinically relevant situation, a mild proteinuric model was chosen: thus, a low amount of adriamycin was used to induce moderate proteinuria, without the development of nephrotic syndrome but with chronic tubular epithelial cell activation and progressive interstitial remodeling. In this model, we chose a therapy aimed at reducing interstitial remodeling, thus not aiming to reduce proteinuria even further, but to target its downstream consequences. Therefore, in this interventional study we specifically blocked lymph vessel (LV) formation (antibody treatment with anti-VEGFR3), monocyte/macrophage influx (clodronate liposomes, which selectively deplete monocytes/macrophages), and lymphocyte and α-SMA-positive-cell influx (by oral FTY720, as an S1P receptor agonist) separately to investigate the role of each of these phenomena in tubulointerstitial remodeling in proteinuric nephropathy. As read-out parameters we evaluated proteinuria and histological changes.

## RESULTS

### Proteinuria developed over time in adriamycin-injected rats

Urinary protein excretion was significantly increased at week 6 in the adriamycin-injected rats compared with their saline-injected controls [146 (ranging from 77 to 230) in adriamycin-injected rats vs 18 (13-27) mg/24 h in control rats; *P*<0.001]. Except for proteinuria, at week 6 other clinical parameters, such as body weight, blood pressure and kidney function, evidenced by creatinine clearance, were not significantly different between healthy and proteinuric rats, but heart rate was reduced in proteinuric rats (Table 1). Also, there was no difference in water intake between both groups of rats at that time. However, food intake was significantly higher in the proteinuria rats (Table 1). In this way, we developed a model of so-called pure proteinuria without signs of nephrotic syndrome.

**Table 1.**
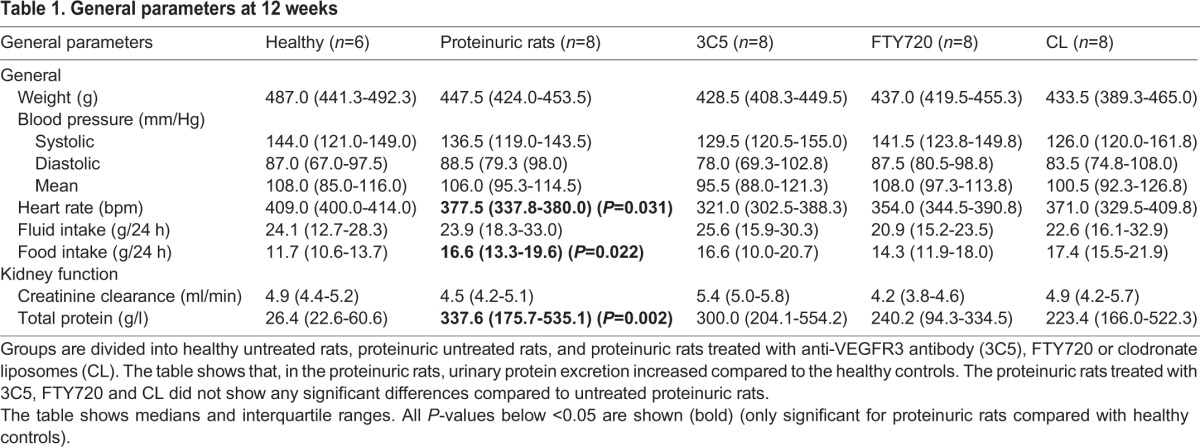
**General parameters at 12 weeks**

**Fig. 1.**
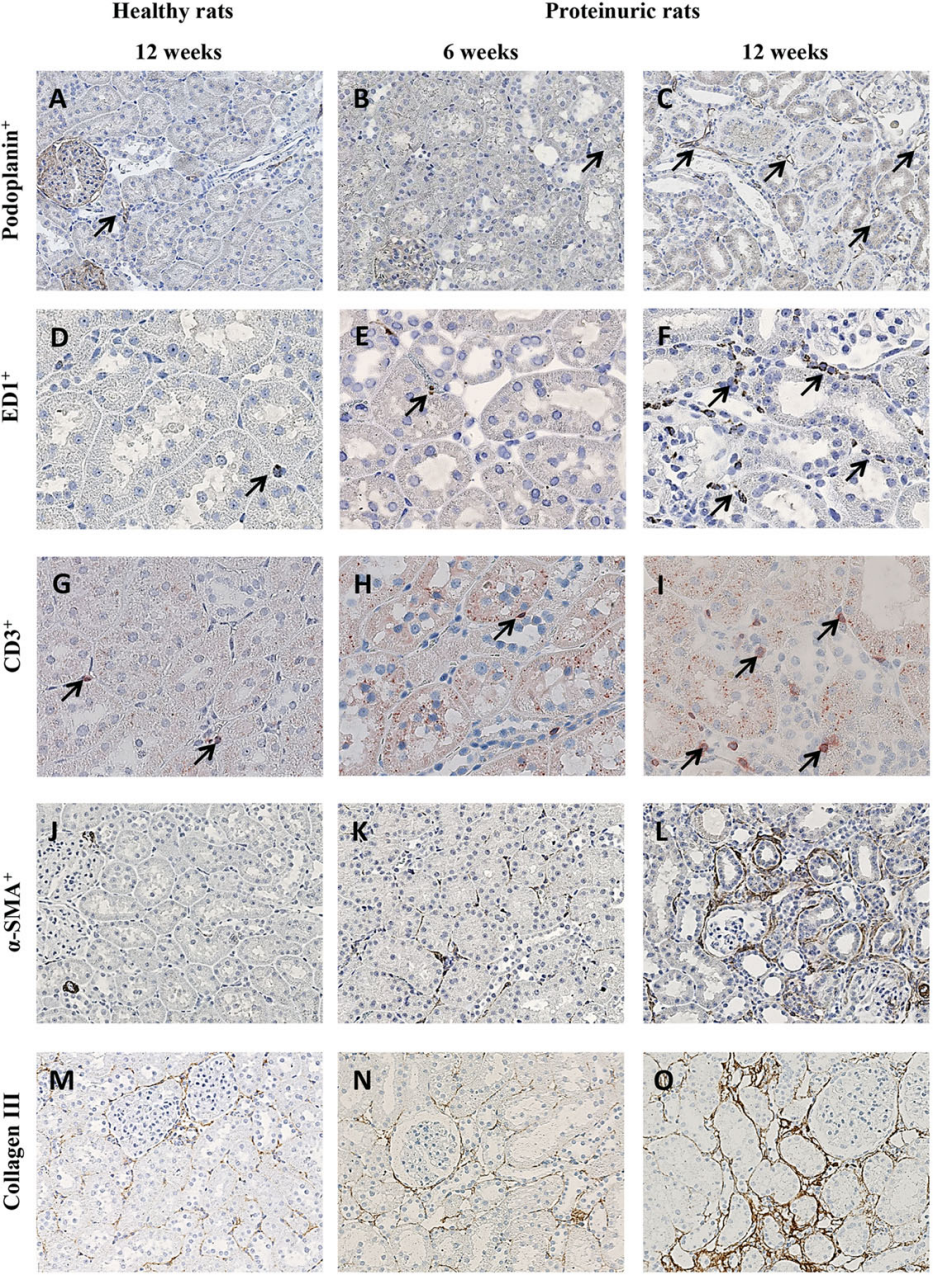
**Development of renal lymphangiogenesis, inflammation and fibrosis in an adriamycin-induced proteinuria model.** Representative photomicrographs show the differences in several markers in the kidneys of healthy rats, and in proteinuric rats at week 6 and week 12. (A-C) Podoplanin+ LVs (arrows); (D-F) ED1+ macrophages (arrows); (G-I) CD3+ T cells (arrows); (J-L) α-SMA+ myofibroblasts; (M-O) collagen III deposition. All markers were significantly increased at week 12 in proteinuric rats compared with the healthy controls at week 12 and the proteinuric rats at week 6. For quantification of these data at 12 weeks between proteinuric rats with and without treatment, see Figs 2, 4 and 6. Magnification: (A-C;J-O) 200×; (D-I) 400×.

**Fig. 2.**
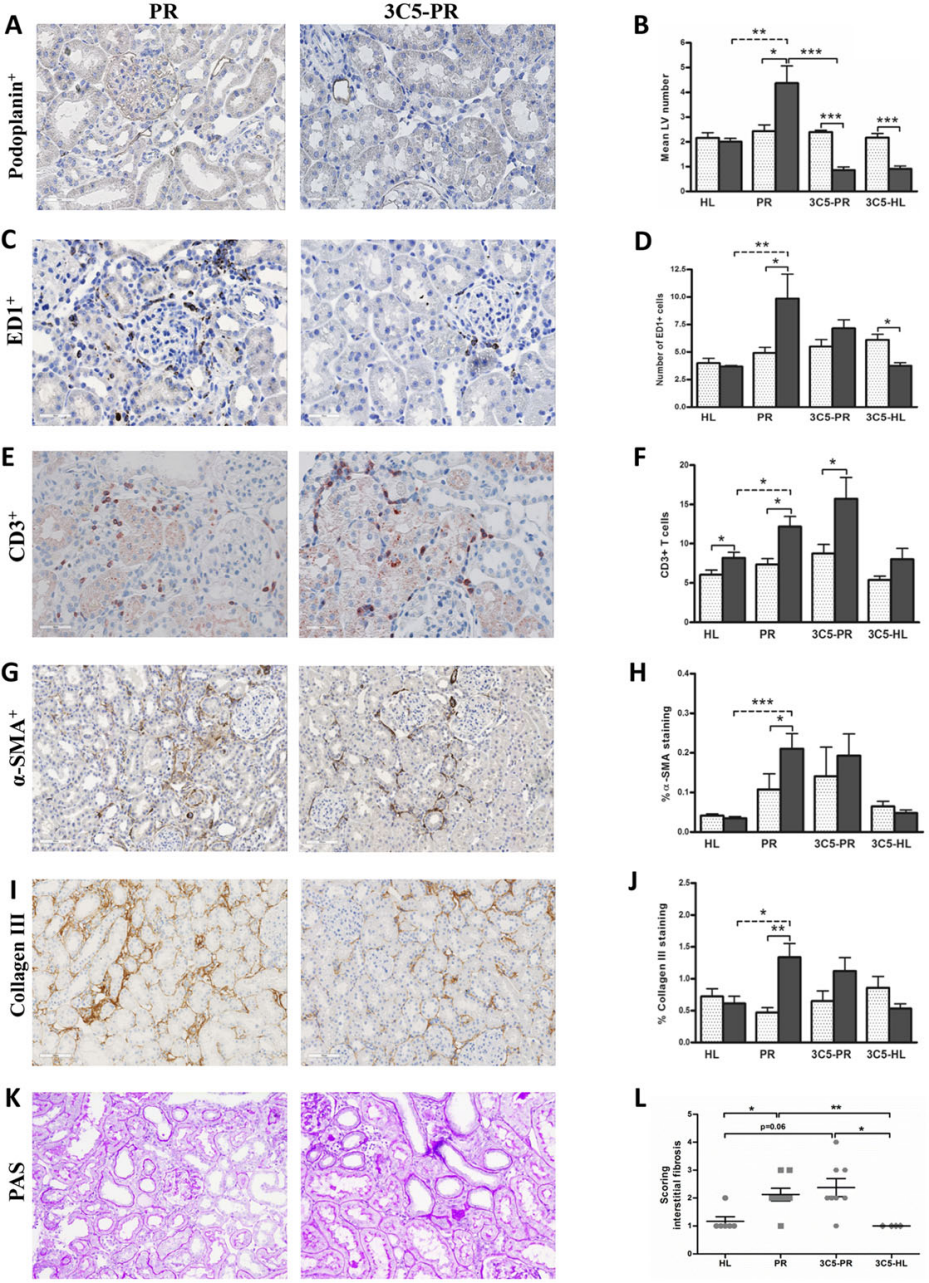
**Effects of anti-VEGFR3 antibody treatment on renal lymphangiogenesis, inflammation and fibrosis.** Quantification of renal cortical podoplanin-positive vessel-like structures of the rats who received treatment with anti-VEGFR3 antibody (IMC-3C5) showed a significant reduction of LV number at 12 weeks (A,B; 400×), whereas it showed a non-significant trend in reducing the number of macrophages in the cortical interstitium of proteinuric rats (C,D; 400×), and did not influence T-cell influx (E,F; 400×) at week 12. Anti-VEGFR3 antibody also did not have a significant effect on α-SMA (G,H; 200×), collagen III deposition (I,J; 200×) and interstitial fibrosis (K,L; 200×). White dotted bars represent week 6 before treatment; black bars represent week 12 after treatment. The PAS staining quantification (interstitial fibrosis) is showed at 12 weeks. HL, healthy; PR, proteinuric untreated; 3C5, anti-VEGFR3 antibody. *P<0.05, **P<0.01, ***P<0.001.

Histological inspection of the kidneys revealed that, at 6 weeks, a non-significant influx of α-SMA-positive cells was observed in adriamycin-injected rats compared with healthy controls. Otherwise, no changes were seen in week-6 proteinuric kidneys, neither for number of LVs, ED-1-positive macrophages and CD3-positive T cells, nor for interstitial fibrosis evidenced by collagen III quantification (Fig. 1) and periodic acid-Schiff (PAS) staining (not shown at week 6). In contrast, at week 12, tubulointerstitial tissue remodeling occurred. This was characterized by lymphangiogenesis measured by an increase in LV density (Fig. 1C), increased numbers of ED-1-positive macrophages (Fig. 1F) and CD3-positive T cells (Fig. 1I), increased α-SMA-positive myofibroblasts (Fig. 1L), and interstitial fibrosis measured by collagen III deposition (Fig. 1O) and PAS staining (Fig. 2K).

### Anti-VEGFR3 antibody totally prevented LV formation in proteinuric rats

**Fig. 3.**
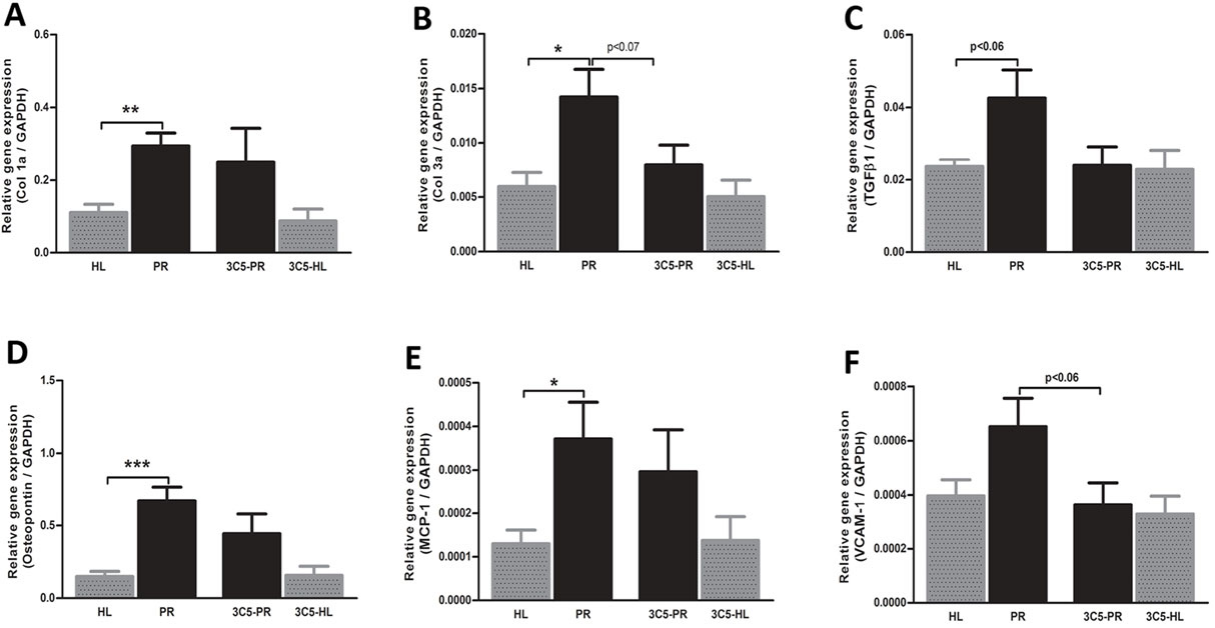
**Effects of anti-VEGFR3 antibody treatment on mRNA expression of fibrotic and inflammatory markers.** Anti-VEGFR3 injections tended to reduce the proteinuria-induced expression of collagen III (α1) and *TGF-β1* mRNA, but not collagen I (α1) (A-C). Likewise, anti VEGFR3 intervention tended to reduce the proteinuria-induced mRNA expression of *MCP-1*, osteopontin and *VCAM-1* (D-F). HL, healthy; PR, proteinuric untreated; 3C5, anti-VEGFR3 antibody. *P<0.05, **P<0.01, ***P<0.001.

Treatment for 6 consecutive weeks with anti-VEGFR3 antibody (IMC-3C5) in proteinuric rats (weeks 6-12) did not significantly alter proteinuria, compared with non-treated proteinuric controls. No noticeable changes were observed in body weight, blood pressure, heart rate, food and fluid intake, and creatinine clearance compared with untreated proteinuric rats (Table 1). On histology, proteinuric kidneys at week 12 presented significantly more podoplanin-positive LVs by immunohistochemistry in the cortical interstitium, compared to week 6, implying renal lymphangiogenesis between weeks 6 to 12 (Fig. 1A-C; *P*<0.05). IMC-3C5 treatment showed a robust and significant decrease in the amount of LVs, not only in the proteinuric rat kidneys, compared with non-treated proteinuric rats (*P*<0.001), but also in the kidneys of healthy rats upon treatment, compared with the non-treated healthy controls (Fig. 2A,B; *P*<0.001). Looking at macrophages and T cells, although the proteinuric rats treated with IMC-3C5 showed a trend in reducing the ED1-positive macrophages, this was not statistically significant (Fig. 2C,D). Rats treated with IMC-3C5 antibody did not show any marked changes in T-cell number (Fig. 2E,F). α-SMA and collagen III expression and interstitial fibrosis (scored by PAS staining) did not show any marked changes after IMC-3C5 treatment (Fig. 2G-L). Regarding mRNA level [quantitative reverse transcriptase polymerase chain reaction (qRT-PCR)], IMC-3C5 treatment decreased the collagen III (α1) mRNA level almost significantly, but not collagen I (α1) and TGF-β1. This intervention did not show significant effects on inflammatory marker vascular cell adhesion molecule 1 (VCAM-1), monocyte chemoattractant protein-1 (MCP-1/CCL2) and osteopontin (Fig. 3), although a tendency towards a reduction of these inflammatory markers was suggestive. There were no significant differences for total white blood count, lymphocytes, neutrophils, basophils and eosinophils in IMC-3C5-treated groups. Thus, treatment of proteinuric rats with anti-VEGFR3 completely prevented tubulointerstitial LV formation, however without prominent changes in tubulointerstitial inflammatory and fibrotic markers.

### FTY720 prevented the increase in myofibroblast accumulation, T-cell infiltration and interstitial fibrosis, but not collagen III deposition, macrophage influx or LV number

**Fig. 4.**
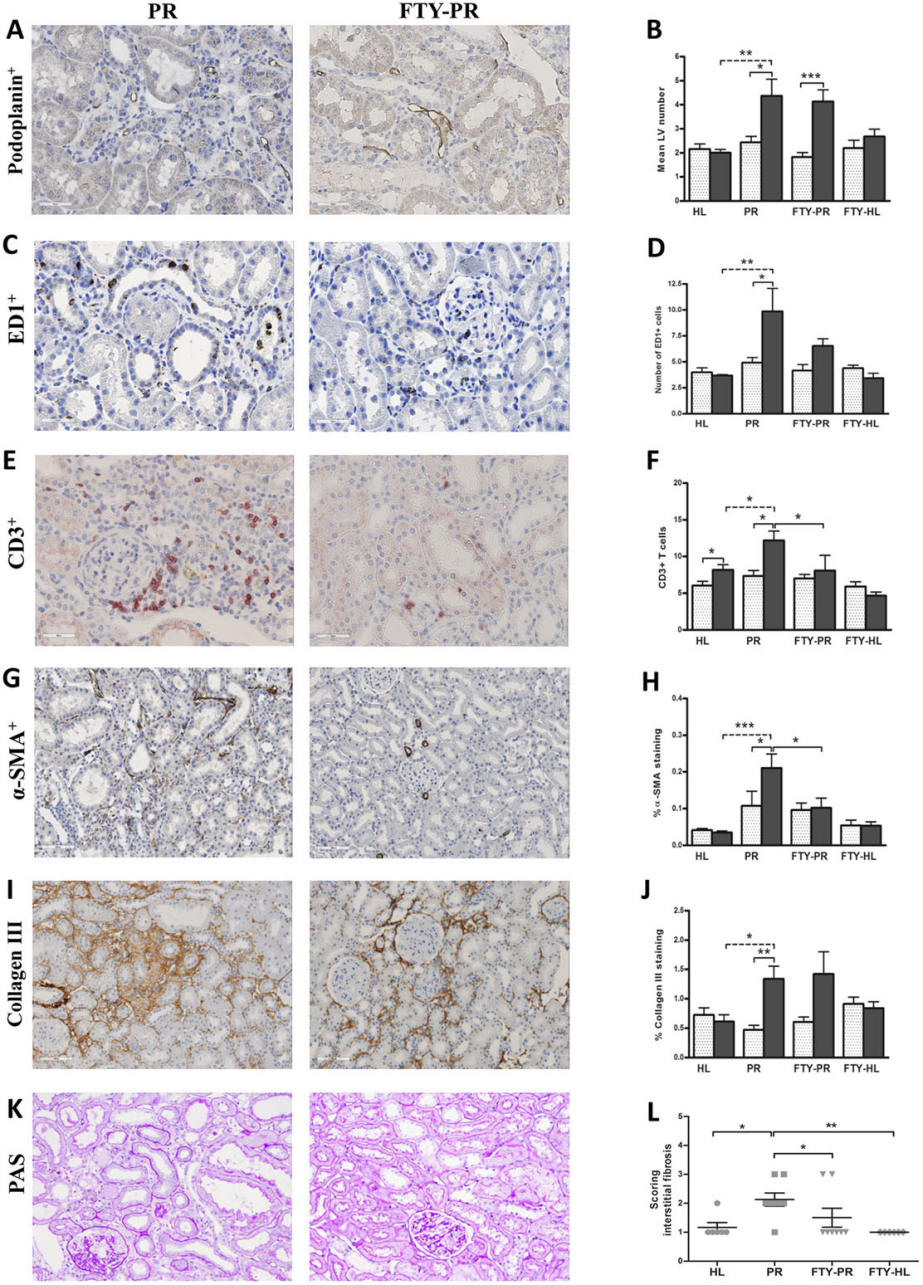
**Effects of FTY720 treatment on renal lymphangiogenesis, inflammation and fibrosis.** Quantification of the staining of kidneys from proteinuric rats treated with FTY720 did not show any effect on the increased number of LVs in proteinuric rats at week 12 (A,B). Although FTY720 did not show a significant effect on the influx of macrophages (C,D), it did show a complete blocking of T-cell influx at week 12 compared with the untreated proteinuric rats (E,F) and also of α-SMA-positive cells at 12 weeks (G,H), whereas it did not show a significant effect on collagen III deposition (I,J). Nevertheless, interstitial fibrosis (PAS scoring) revealed that FTY720 could markedly prevent the development of interstitial fibrosis in proteinuric rats compared with healthy controls at week 12 (K,L). White dotted bars represent week 6 before treatment; black bars represent week 12 after treatment. PAS staining and quantification has been done at week 12. HL, healthy; PR, proteinuric untreated; FTY, FTY720. *P<0.05, **P<0.01, ***P<0.001.

**Fig. 5.**
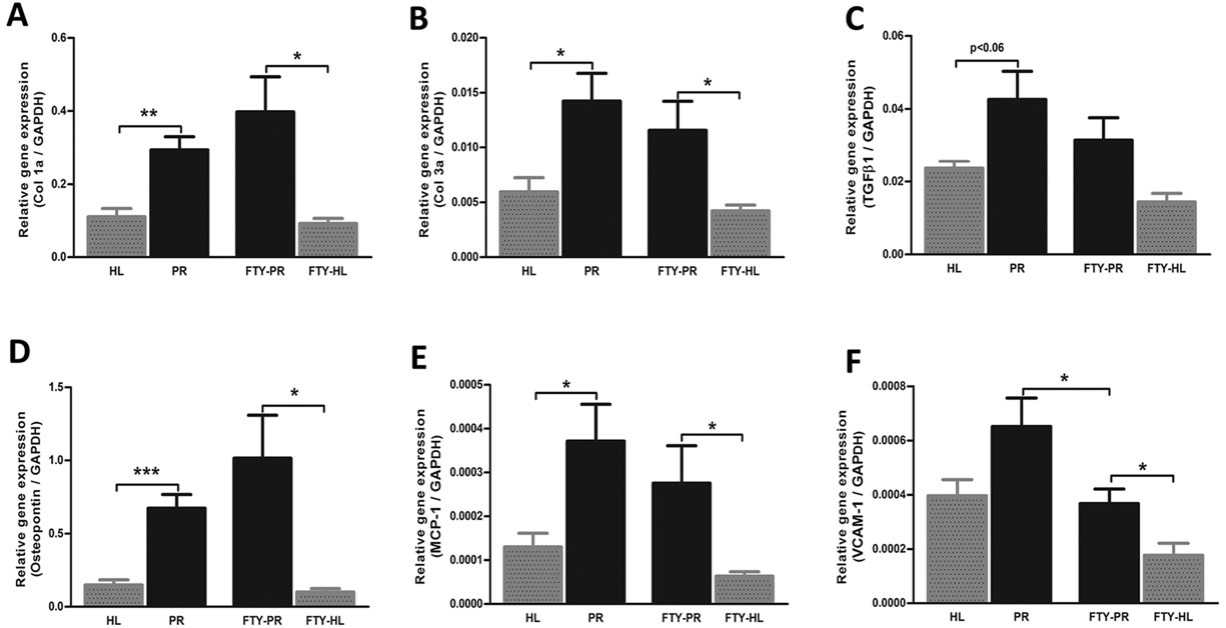
**Effects of FTY720 treatment on mRNA expression of fibrotic and inflammatory markers.** Quantitative RT-PCR data showed that the mRNA expression of fibrotic and inflammatory markers, which have been increased significantly upon proteinuria (A-F), was not reduced by FTY720 treatment (A-E), except for significant prevention of *VCAM-1* mRNA increase (F). HL, healthy; PR, proteinuric untreated; FTY, FTY720. *P<0.05, **P<0.01, ***P<0.001.

Treatment of proteinuric rats with FTY720 did not affect body weight, food and water intake, blood pressure, heart rate, creatinine clearance or proteinuria (Table 1). FTY720 treatment had no effect on renal lymphangiogenesis (Fig. 4A,B). The influx of ED1-positive macrophages showed a tendency to be reduced upon FTY720 treatment, although not significantly (Fig. 4C,D). Because the number of white blood cells and leukocytes were strongly reduced by FTY720 treatment (Fig. 8A,B; *P*<0.001), renal influx of CD3-positive T cells was significantly reduced at 12 weeks (Fig. 4E,F; *P*<0.05). FTY720 completely prevented α-SMA-positive myofibroblast accumulation at week 12 compared with non-treated proteinuric rats (Fig. 4G,H; *P*<0.05), but did not show any marked effect on collagen III deposition by immunohistochemistry (Fig. 4I,J). Nevertheless, this intervention significantly reduced interstitial fibrosis scored by PAS staining (Fig. 4K,L; *P*<0.05). At the mRNA level, FTY720 also could not prevent the increase in collagen I (α1), collagen III (α1), *TGF-β1*, *MCP-1/CCL2* and osteopontin, although significantly prevented the increase of *VCAM-1* mRNA expression (Fig. 5). In summary, in the kidneys of FTY720-treated proteinuric rats, accumulation of α-SMA-positive myofibroblasts, CD3-positive T cells and interstitial fibrosis were prevented; however, there was no effect on collagen III deposition, macrophage influx and lymphangiogenesis.

### Clodronate liposome prevented macrophage influx in the kidney without any major effect on other histological and clinical parameters

**Fig. 6.**
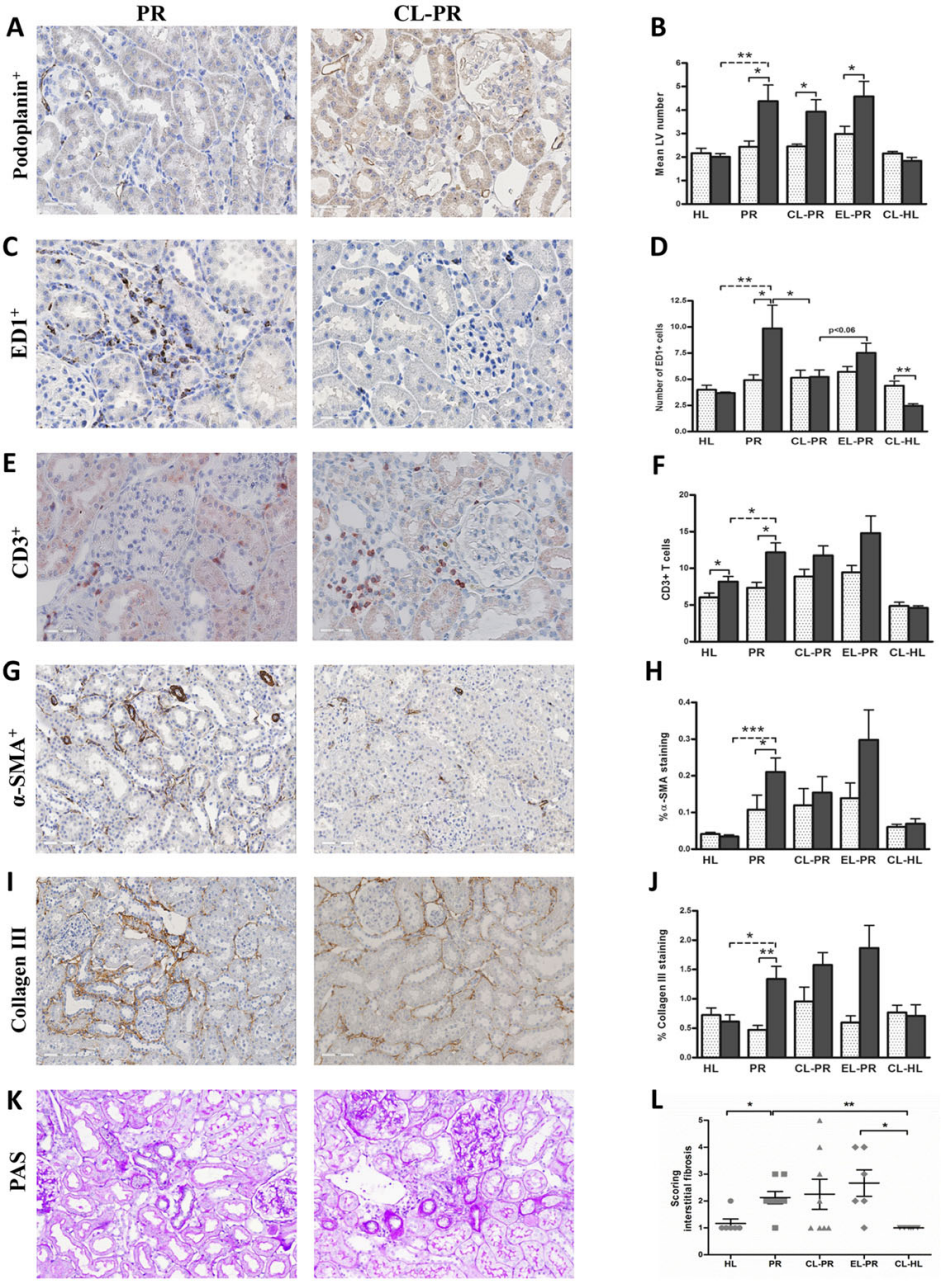
**Effects of macrophages depletion by CLs on renal lymphangiogenesis, inflammation and fibrosis.** Quantification of immunohistochemical stainings of the kidneys of CL-treated proteinuric rats did not prevent the increase in LV number in proteinuric rats (A,B) despite completely blocking macrophage influx at week 12 (C,D). However, this treatment had no obvious effect on T-cell influx (E,F), and did not have a significant effect on α-SMA (G,H) or collagen III deposition (I,J). In the same line, the development of interstitial fibrosis was also not inhibited by CL intervention (K,L). White dotted bars represent week 6 before treatment; black bars represent week 12 after treatment. The PAS staining quantification (interstitial fibrosis) is shown at 12 weeks. HL, healthy; PR, proteinuric untreated; CL, clodronate liposome; EL, empty liposomes. *P<0.05, **P<0.01, ***P<0.001.

**Fig. 7.**
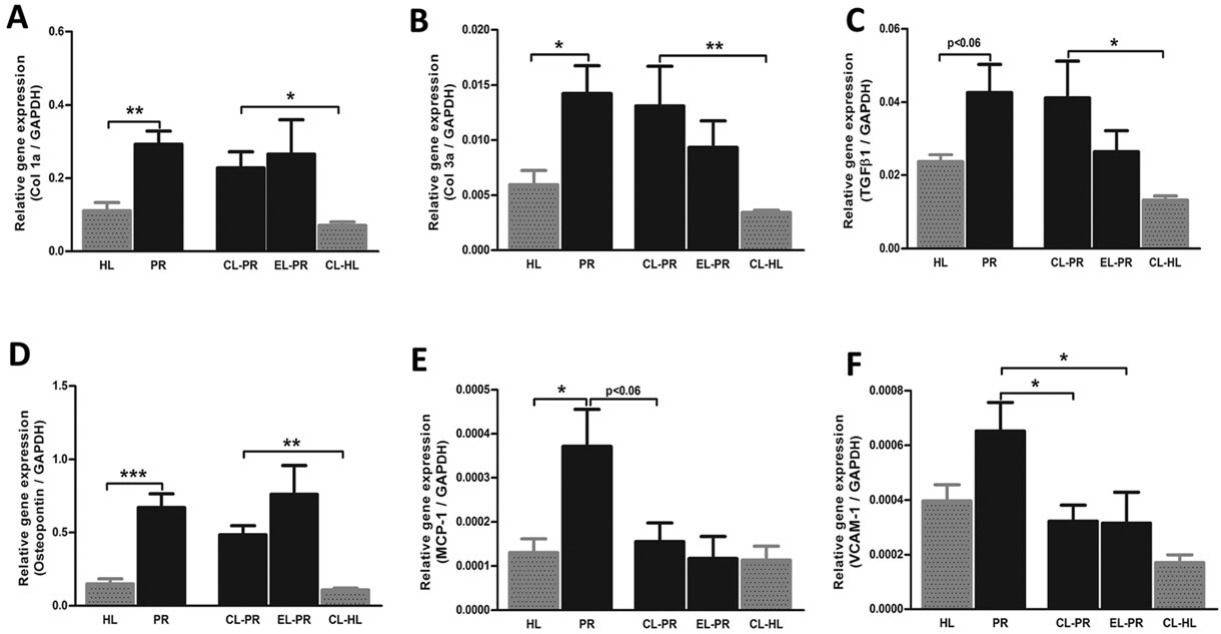
**Effects of CL treatment on mRNA expression of fibrotic and inflammatory markers.** Targeting macrophages by CLs did not markedly alter the mRNA expression of the fibrotic markers collagen I (α1), collagen III (α1) and *TGF-β1* compared with non-treated proteinuric rats (A-C). The CL intervention strongly prevented the increase of *VCAM-1* mRNA expression in proteinuric rats (F). *MCP-1* expression was non-significantly (*P*<0.06) reduced by CL treatment (E), whereas osteopontin expression was not influenced by the treatment (D). HL, healthy; PR, proteinuric untreated; CL, clodronate liposome; EL, empty liposome. *P<0.05, **P<0.01, ***P<0.001.

**Fig. 8.**
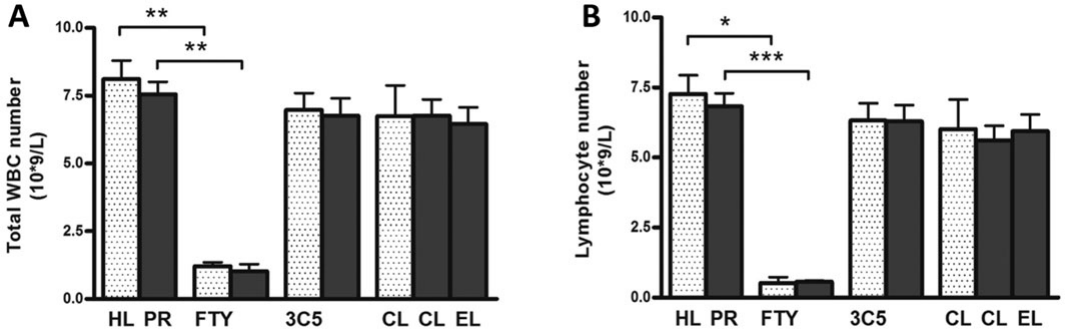
**Number of total white blood cells (WBCs) and lymphocytes in the blood in both healthy and proteinuric rats at week 12.** Total WBCs did not show any difference in healthy non-proteinuric compared to proteinuric rats (A). However, upon treatment with FTY720, the total WBCs dramatically decreased at week 12, in both healthy (white dotted bars) and proteinuric (black bars) groups. CL and IMC-3C5 did not show any effect. In order to see the effect of treatments on specific subsets of WBCs in the blood, we also measured the number of lymphocytes. FTY720 markedly reduced the number of lymphocyte in the circulation, even in healthy rats who received the drug. However, again the other two interventions did not have any effect on lymphocyte number in the blood circulation (B). HL, healthy; PR, proteinuric untreated; CL, clodronate liposome; EL, empty liposome; 3C5, anti-VEGFR3 antibody; FTY, FTY720. *P<0.05, **P<0.01, ***P<0.001.

Targeting monocyte/macrophages by clodronate liposomes (CLs) in proteinuric rats did not result in changes in body weight, blood pressure, heart rate, food and water intake, creatinine clearance and proteinuria (Table 1). This treatment also did not prevent the formation of new LVs in proteinuric rats compared with non-treated proteinuric control rats (Fig. 6A,B). However, kidneys of proteinuric rats showed a significant decrease in macrophage number upon CL treatment (Fig. 6C,D; *P*<0.05), whereas proteinuric rats treated by ‘placebo’ liposomes (PBS instead of clodronate) showed a small non-significant reduction (Fig. 6D). Even in non-proteinuric healthy controls treated with CLs, the number of kidney macrophages significantly decreased compared with non-treated healthy controls (Fig. 6D; *P*<0.01). CL treatment did not influence the number of circulating white blood cells and lymphocytes in the blood (Fig. 8). Treatment of proteinuric rats with CLs, despite effective prevention of macrophage influx, did not significantly influence influx of T cells, myofibroblast accumulation, interstitial fibrosis or collagen III deposition (Fig. 6E-L). At the mRNA level, this treatment also did not show any marked effect on collagen I (α1), collagen III (α1), *TGF-β1* and osteopontin, whereas it inhibited the increase in mRNA expression of *MCP-1/CCL2* and *VCAM-1* (Fig. 7). Thus, despite effective reduction of renal inflammation by CL treatment, interstitial fibrosis and lymphangiogenesis was not influenced by this intervention.

## DISCUSSION

In the adriamycin-induced proteinuria model, we targeted tubulointerstitial lymphangiogenesis (VEGFR3 blockade), monocyte/macrophage influx (depletion by CLs), and pre-fibrotic myofibroblast accumulation and interstitial fibrosis (by the S1P agonist FTY720). Anti-VEGFR3 antibody completely blocked renal lymphangiogenesis in proteinuric rats. Nevertheless, on a histological level, the anti-VEGFR3 antibody did not show any major effects on inflammatory (macrophages and T cells) or fibrotic (α-SMA, collagen III and interstitial fibrosis) markers despite some apparent reductions in fibrotic and inflammatory markers at the mRNA level. FTY720 considerably prevented α-SMA-positive myofibroblast accumulation and interstitial fibrosis, but not collagen III deposition and lymphangiogenesis. The treatment of proteinuric rats with CL prevented the increase in tissue macrophage number in proteinuric kidneys, but did not show major changes on the clinical parameters, neither on tubulointerstitial lymphangiogenesis nor fibrotic markers. This study thereby shows the dissociation of inflammatory (macrophages, T cells) or fibrotic (myofibroblasts, collagen III and interstitial fibrosis) responses from renal lymphangiogenesis, at least in this proteinuric-nephropathy model. Importantly, proteinuria-induced interstitial fibrosis cannot be halted by blocking lymphangiogenesis or the influx of macrophages.

Tubulointerstitial remodeling is one of the key events in proteinuric nephropathy, which in the end causes massive renal fibrosis culminating into loss of renal function and ESRD (Nangaku, 2004; Theilig, 2010). Although targeting proteinuria directly is the most commonly used and effective treatment in the clinic, complete annihilation of proteinuria is very difficult and attempts to do so resulted in increased mortality (Nangaku, 2004; Khan et al., 2013; Hou et al., 2007; Chen et al., 2014). Therefore, preventing and/or curing damage downstream of proteinuria in the kidney would be of high importance in order to preserve kidney function, and halt progression towards CKD and eventually ESRD.

Vascular remodeling plays a major role in tubulointerstitial homeostasis in the microenvironment of all organs, and specifically in the kidney (Rienstra et al., 2010; Schrijvers et al., 2004). Lymphangiogenesis, the growth and formation of new LVs, has caught increasing attention over the last years owing to its immense importance in many pathological conditions in the body (Alitalo, 2011). Lymphangiogenesis has been shown to be in close relation to fibrogenesis in different organs, including the kidney (Sakamoto et al., 2009; Zampell et al., 2012; El-Chemaly et al., 2009). Yet, the causal interplay between fibrosis and lymphangiogenesis has not been clearly explored. Meinecke et al. showed in an elegant study that LVs play a central role in fibrogenesis, at least in pulmonary fibrosis (Meinecke et al., 2012). They meticulously showed that, in the early stage of the disease, activated LECs stimulate PDGFR-β-expressing mural cells by secretion of platelet derived growth factor-B (PDGF-B), recruiting these cells around LVs and then by attaching to LVs, impeding their drainage capacity. These defects then hamper the most important function of LVs, which is fluid drainage, and thereby lead to fibrotic processes in the lung. Although it is not yet known whether this phenomenon holds true in renal fibrosis, this study proposed an important role of LVs in fibrogenesis. CCL21, by signaling CCR7-receptor-expressing cells, initiates a vital pathway in renal fibrogenesis (Sakai et al., 2006). LECs of renal LVs are also able to secrete CCL21 (Kerjaschki et al., 2004). Because blocking of lymphangiogenesis by anti-VEGFR3 decreases CCL21 secretion (Nykänen et al., 2010), this strategy might be useful in targeting fibrosis. We previously showed that, in the experimental unilateral proteinuric model, prolonged and sustained proteinuria triggers new LV formation in the kidney and that renal lymphangiogenesis occurs before the influx of macrophages and collagen deposition (Yazdani et al., 2012). To explore the importance of LVs in tubulointerstitial damage and its potential roles in modulating the microenvironmental milieu, we targeted lymphangiogenesis specifically. Anti-VEGFR3 antibodies have been extensively studied in experimental models, and are currently in Phase 1 clinical trial for the treatment of advanced malignant tumors (http://clinicaltrials.gov/ct2/show/NCT01288989). Therefore, in this current study, by specific blocking of lymphangiogenesis in a renal injury model, we aimed to explore more about its involvement in this disease. In this study, IMC-3C5 completely blocked lymphangiogenesis in our proteinuric model in rats and also in the healthy controls, which indicates LV regression (Mäkinen et al., 2001). This intriguing observation needs further studies to see what could be the consequence of this decrease in LV number, such as edema formation or delaying exit of inflammatory cells, etc. This intervention, however, failed in preventing renal inflammation (at least not macrophage and T-cell number) and fibrosis (at least not α-SMA and collagen III expression, and interstitial fibrosis scored by PAS staining), although some apparent effects on mediators of inflammation and fibrosis on mRNA level is suggested by qRT-PCR data. The reason might be that, in this model, tubular epithelial cells, which continuously encounter the ultrafiltrated plasma proteins and are being activated, are the main source of many chemokines and mediators that induce tubulointerstitial remolding such as lymphangiogenesis, inflammation and fibrosis (Moreno et al., 2014).

LVs are an integral part of the inflammatory process, and they have been proposed as a therapeutic target in inflammation (Johnson and Jackson, 2010; Dieterich et al., 2014; Kim et al., 2012). Different leukocytes are capable of prompting lymphangiogenesis (Loffredo et al., 2014); however, among them, the role of macrophages is far more highlighted (Ran and Montgomery, 2012; Kerjaschki, 2005), and macrophage depletion or reduction has been shown to abolish lymphangiogenesis in several disease models (Poosti et al., 2012; Maruyama et al., 2012). Still, the role of macrophages, specifically in renal lymphangiogenesis, is not distinguished clearly. Several groups, including ours, have shown that macrophages are actively involved in inducing lymphangiogenesis in kidney diseases (Yazdani et al., 2014); however, there are some conflicting findings in these studies. Lee et al. (2013) depleted macrophages by CLs in unilateral ureteral obstruction (UUO) kidney damage in mice and found the striking blockage of lymphangiogenesis. In a rat UUO model, Suzuki et al. (2012) showed tubular epithelial cells to be the main inducer of renal lymphangiogenesis. It seems that, at least in this experimental model, macrophages are not the main lymphangiogenic inducer. We now showed that complete prevention of tubulointerstitial macrophage influx also reduced some markers of inflammation at the mRNA level. However, neither interstitial fibrosis nor lymphangiogenesis could be reduced by this intervention. Hence, the role of macrophages in inducing lymphangiogenesis seems to be very much context-dependent.

FTY720, an FDA-approved drug to treat multiple sclerosis, exerts different kinds of effects in the body (Halmer et al., 2014; Pitman et al., 2012). One of the most well-known influences is the immunosuppression by blocking the egress of lymphocytes from the lymph nodes, thereby reducing inflammation (Kabashima et al., 2006; Matloubian et al., 2004). Several reports have shown the beneficial effect of FTY720 not only in a renal inflammatory reactions, but also in hampering renal profibrotic and fibrotic development, e.g. myofibroblast activation and collagen deposition (Shiohira et al., 2013; Ni et al., 2013a,b). By binding to the S1P1 receptor on LECs, FTY720 proved to be an effective drug in blocking lymphangiogenesis (Yin et al., 2011). In this current study, FTY720 treatment effectively reduced the number of lymphocytes in the blood circulation, and T cells in proteinuric kidneys, but did not have any impact on renal lymphangiogenesis. Interestingly, although FTY720 significantly prevented the increase of α-SMA-positive myofibroblasts, it was not effective in decreasing collagen III deposition, although it significantly reduced tubulointerstitial fibrosis (PAS scoring). Apparently, collagen III is not fully representative for interstitial fibrosis, which is a reflection of interstitial matrix accumulation of many different extracellular matrix molecules. Results also showed that α-SMA is not an ideal marker for collagen-secreting cells in interstitial injuries, because many (myo)fibroblasts that do not express α-SMA are able to deposit collagen (Boor et al., 2010; Farris and Colvin, 2012; Hinz et al., 2012).

In summary, our study showed that tubulointerstitial fibrosis, inflammation and lymphangiogenesis are rather independent tissue remodeling responses, at least under proteinuric conditions. Our work also shows that blocking renal interstitial lymphangiogenesis or inflammation did not effectively reduce the development of renal fibrosis. It proposes that, for the treatment of the downstream consequences of proteinuria, there is no specific target in just one of the tubulointerstitial changes that we investigated in this study. Data rather suggest a combination of intervention strategies to reduce proteinuria-driven tubulointerstitial tissue remodeling is required, e.g. by combining FTY720 or CLs with lymphangiostatic treatments to evaluate the effects on fibrosis and functional renal outcome parameters. Earlier data indicated that activated tubular epithelial cells trigger lymphangiogenesis, inflammation and fibrosis. Our group previously showed that specific targeting of the Rho-kinase pathway in proximal tubular epithelial cells markedly reduced renal inflammation and renal lymphangiogenesis in an acute renal allograft rejection model (Poosti et al., 2012). Although it warrants future studies, these findings suggest that strategies to preserve tubular epithelial cells or directly target their secreted chemokines and mediators could be a promising approach in preventing or treating tubulointerstitial damages secondary to proteinuria.

## MATERIALS AND METHODS

### Animal experimental protocol and treatments

Proteinuria was induced in 78 3-month-old male Wistar rats (weighing 180-200 g) by single injection of adriamycin in the tail vein [1.8 mg/kg body weight (BW)], and healthy rats served as controls. After 6 weeks, when proteinuria had developed (∼150 mg/24 h), a kidney biopsy was taken via dorsolateral incision. After recovery, 60 rats were assigned randomly to one of the interventional or control groups. 18 rats did not develop sufficient proteinuria and were excluded from the study. Proteinuric rats were randomly divided into five groups: a proteinuric untreated group (*n*=8), and four interventional groups, which were treated with FTY720 (*n*=8), anti-VEGFR3 antibody (*n*=8), CLs (*n*=8) or empty (PBS) liposomes (*n*=6), which served as a control for the CLs. The healthy rats were randomly divided into a healthy untreated control group (*n*=6) and three different healthy control groups, which were treated with FTY720 (*n*=6), anti-VEGFR3 antibody (*n*=4) or CLs (*n*=6). The treatment by the above-mentioned agents then started from week 6 and was continued until week 12: anti-VEGFR3 antibody (IMC-3C5, ImClone/Eli Lilly, USA) intraperitoneally (i.p.) 40 mg/kg BW, three times per week; CLs (ClodronateLiposomes.org, The Netherlands) i.p. twice weekly 1 ml/rat; and FTY720 (Novartis, Basel, Switzerland) 1 mg/kg BW/day in drinking water. At week 12, blood pressure was measured under general anesthesia with the Cardiocap/5 (Datex-Ohmeda, Newark, USA). Then, after saline perfusion, organs were harvested and some parts were preserved in liquid nitrogen for cryosections, and other parts in formaldehyde 10% for paraffin embedment.

At the beginning of the study, at the time of the biopsy (6 weeks) and at the end of the experiment (12 weeks), body weight was measured, blood samples were collected and rats were placed in metabolic cages for 24 h for urine collection and the measurement of food and water intake. Proteinuria was determined in urine samples by a turbidimetric assay (Roche Modular P, Mannheim, Germany). Experimental procedures were carried out according to the national guidelines for the care and use of laboratory animals, and approved by the local Animal Ethics Committee of the University of Groningen.

### Immunohistochemistry

Staining was performed on 3-μm-thick formalin-fixed paraffin sections after deparaffinization in xylene and rehydration in alcohol series. Antigen retrieval was done for 15 min in a microwave oven for Tris/EDTA buffer pH:9 and citrate buffer pH:6, or overnight at 80°C in Tris/HCl buffer pH:8. Endogenous peroxidase activity was blocked with 0.3% hydrogen peroxide. Sections were incubated for 1 h or overnight at 4°C with the following primary antibodies: mouse anti-human α-SMA (clone 1A4, Sigma-Aldrich, St Louis, USA), goat anti-collagen III (cat. no. 1330-01, SouthernBiotech, Birmingham, USA), rabbit anti-rat CD3 (clone A0452, Dako, Glostrup, Denmark), mouse anti-rat CD68 (clone ED1, AbD Serotec, Oxford, UK) and mouse anti-rat podoplanin (cat. no. 11-035, Angio Bio, Del Mar, USA). After this step, the sections were incubated with secondary and tertiary antibodies diluted in PBS/BSA 1% and 1% normal rat serum. We used rabbit anti-mouse Ig horseradish peroxidase (HRP), goat anti-rabbit Ig HRP, goat anti-mouse Ig HRP, rabbit anti-goat Ig HRP, swine anti-rabbit HRP and anti-rabbit poly HRP (all from Dako, Glostrup, Denmark). As negative controls, the primary antibodies were replaced by PBS/BSA 1%. Bound antibodies were visualized by aminoethylcarbazole (AEC) or by the peroxidase substrate 3,3′-diaminobenzidine (DAB) (Sigma-Aldrich, St Louis, USA) and then counterstained with diluted hematoxylin. PAS was also performed on series of sections in order to quantify the extent of structural changes (interstitial fibrosis). The sections were then scanned by a NanoZoomer HT (Hamamatsu Photonics K.K., Shizuoka Pref., Japan). The quantification was done using Aperio ImageScope software (version 9.1.772.1570, Aperio Technologies Inc., Vista, CA, USA) and ImageJ 1.46r (Rasband, W.S., U.S. National Institutes of Health).

### Quantification of LVs and renal histomorphology

For identification of LVs, we counted podoplanin-positive vessels in 30 cortical interstitial fields per kidney. The amount of collagen III, myofibroblasts (α-SMA), ED1-positive macrophages and CD3-positive T cells were measured as described previously (Yazdani et al., 2012). In short, collagen III expression, myofibroblasts, ED1-positive macrophages and CD3-positive T cells were evaluated in 30 (high or medium power field) cortical interstitial areas of each kidney in a blinded fashion. The quantification was done using ImageJ 1.41 (Rasband, W.S., U.S. National Institutes of Health). PAS staining was semi-quantitatively scored on a scale ranging from 1 to 5. The scoring indicates which part of renal cortical tissue was affected by tubulointerstitial fibrosis (broadening interstitial area in between the tubules): score 1: <1%; score 2: 1-5%; score 3: 6-10%; score 4: 11-20%; score 5: 21-50%.

### Urine and plasma analysis

The sodium, potassium and chloride concentrations in both plasma and urine were analyzed by an electrolyte analyzer ISE (Roche Modular P, Mannheim, Germany). The urea and creatinine in plasma and urine were measured by an enzymatic UV assay (Roche Modular P). For the measurement of total protein in plasma/serum, we used a colorimetric assay (Roche Modular P).

### RNA isolation and cDNA synthesis

For RNA isolation from kidney tissue, we used the Favorprep RNA minikit (Favorgen Biotech Corp., Denmark). For each sample, we used 5-µm sections, in total weighing approximately 30 mg (no DNase treatment during RNA isolation). Concentration measurement was done by Nanodrop and the integrity of the RNA was tested by running the samples on a 1% agarose gel in loading buffer. cDNA was synthesized using a QuantiTect Reverse Transcription Kit (Qiagen, Germany). Genomic DNA was eliminated during this procedure.

### RT-PCR

mRNA expression of osteopontin, *MCP-1* (*CCL2*), *VCAM-1*, collagen I (α1), collagen III (α1) and *TGF-β1* were determined by qRT-PCR. Primers were bought from QIAGEN, The Netherlands. Primers were: collagen I (α1) primer (5′-AGCCTGAGCCAGCAGATTGA-3′ and 5′-CCAGGTTGCAGCCTTGGTTA-3′), MCP-1 primer (5′-CCGACTCATTGGGATCATCTT-3′ and 5′-TGTCTCAGCCAGATGCAGTTAAT-3′). Other primers were ordered ‘on demand’ with the following order names: Rn_Col3a1_2_SG QuantiTect primer assay (QT01083537), Rn_Tgfb1_1_SG QuantiTect primer assay (QT00187796), Rn-Vcam1-1-SG QuantiTect primer assay (QT00178500) and Rat-Spp-1 RT2 qPCR Primer assay (osteopontin) (PPR44222B).

qRT-PCR was performed using the C1000 CFX384 from Bio-Rad, using SYBR Green (SensiMix SYBR No-ROX kit, GC biotech). *GAPDH* was used as a housekeeping gene to normalize mRNA expression. GAPDH primers: (5′-CATCAAGAAGGTGGTGAAGC-3′ and 5′-ACCACCCTGTTGCTGTAG-3′). 6.7 ng cDNA per well were brought on a 384-well plate (plateHard-Shell PCR plates, 384-well white well/CRL shell). Every sample was measured in triplicate. The cycle procedure was as followed: 10 min at 95°C, with 40 repeats of a 15 s denaturation step at 95°C and a 15 s extension and annealing step at 60°C, followed with a 5 s extension step at 72°C. A dissociation stage was added to ensure that the desired amplification was detected. Results are expressed as 2-deltaCT, and finally presented as relative expression to GAPDH.

### White blood cell counting

The number of white blood cells, neutrophils, lymphocytes, monocytes, eosinophils and basophils were measured by the Sysmex XN9000 (Kobe Japan).

### Statistical analysis

Statistical analyses were performed using SPSS 20.0 (SPSS Inc., Chicago, IL, USA), and GraphPad Prism 5.0 (GraphPad Software Inc., La Jolla, CA, USA) was used for making graphs and figures. Statistical differences were tested using Mann–Whitney *U*-test. Because the PAS staining was scored semi-quantitatively into five categories, by Chi-square analyses we compared the number of kidneys without interstitial fibrosis (score 1) with those showing interstitial fibrosis (score >1). *P*<0.05 was considered statistically significant.
